# Timing influences detection of anti-nephrin antibodies in recurrent FSGS

**DOI:** 10.1172/JCI212285

**Published:** 2026-11-16

**Authors:** Marie-Camille Lafargue, Leonardo V. Riella

**Affiliations:** 1 Center for Transplantation Sciences, Department of Surgery, Massachusetts General Hospital, Boston, Massachusetts, USA.; 2 Ecole Doctorale BioSPC, Université Paris Cité, Paris, France.; 3 Harvard Medical School, Boston, Massachusetts, USA.

**Keywords:** Autoimmunity, Nephrology, Autoimmune diseases, Immunoglobulins, Organ transplantation

**To the Editor:** We read with great interest the study by Pecoraro et al. examining anti-nephrin antibodies in primary and posttransplant podocytopathies (1). The authors should be commended for their careful assessment of assay specificity, particularly the demonstration of α-Gal–mediated cross-reactivity with murine cell–derived antigens. However, several features of the transplant cohort warrant consideration when interpreting the reported lack of association between anti-nephrin antibodies and posttransplant focal segmental glomerulosclerosis (FSGS) recurrence.

First, the timing of recurrence appears atypical for classical recurrent FSGS. Recurrence generally occurs early after transplantation, often within days to weeks, consistent with a circulating factor–mediated process (2). In contrast, among adults sampled during recurrence in Pecoraro et al., the mean time from transplantation to recurrence was 569 days, with substantial variability. As the authors acknowledge, this wide range suggests biological heterogeneity and raises the possibility that some late events may differ mechanistically from classical early recurrent FSGS.

Equally important is the timing of biospecimen collection. Among 34 patients with posttransplant recurrence, only 5 were sampled before transplantation and 7 during recurrence; the remaining 22 were evaluated later after transplantation, including patients with functioning grafts and those who had returned to dialysis. Consequently, few samples interrogated the periods most informative for determining whether anti-nephrin antibodies predict or accompany recurrence. Posttransplant immunosuppression and treatment may further alter antibody levels. Thus, cross-sectional measurements obtained substantially before or after recurrence may not exclude a transient pathogenic autoantibody response.

Prior studies underscore the importance of timing, disease activity, and patient selection. Hengel et al. found the highest anti-nephrin antibody prevalence in patients with active disease before immunosuppressive treatment (3). Shirai et al. reported high rates of anti-nephrin positivity in a predominantly pediatric cohort with early recurrence (4), while Batal et al. associated pretransplant anti-nephrin antibodies with recurrent podocytopathy (5). Together, these observations suggest that both sampling time and clinical phenotype may influence antibody detection.

In this context, our recent multicenter analysis from the TANGO Consortium provides a complementary perspective. Among 136 kidney transplant recipients with biopsy-proven primary FSGS, anti-nephrin antibodies were detected in 11 patients (8%). Although uncommon, antibody positivity identified a subgroup at significantly higher risk of posttransplant recurrence compared with antibody-negative patients (73% vs. 38%; HR 2.62, 95% CI 1.23–5.55; *P* = 0.012) (our unpublished observations). Antibody-positive patients were also significantly younger at disease diagnosis (median 7 vs. 26 years; *P* = 0.003), suggesting that age may contribute to the heterogeneity observed across cohorts. Notably, the majority of antibody-positive patients developed recurrence within the first 2 months following transplantation (1 month [IQR 0.1–2.4]) consistent with a circulating factor–mediated process. In addition, antibody levels tracked with disease activity in available longitudinal samples. These findings suggest that anti-nephrin antibodies are not a universal biomarker across podocytopathies but may instead define a biologically meaningful subset of patients with early, immune-mediated recurrence.

Taken together, differences in patient selection, timing of recurrence, and timing of sample collection may contribute to the apparent discrepancies between studies (Table 1). Rather than excluding a role for anti-nephrin antibodies, the available data support a more nuanced interpretation in which these antibodies may be relevant within a specific clinical context, particularly early posttransplant recurrence.

We agree that assay standardization is essential. Equally important are prospective studies focused on well-defined primary FSGS with systematic sampling before transplantation and during early recurrence. Such studies will determine whether anti-nephrin and other podocyte-directed autoantibodies can ultimately inform risk stratification and targeted therapy.

## Figures and Tables

**Table 1:**
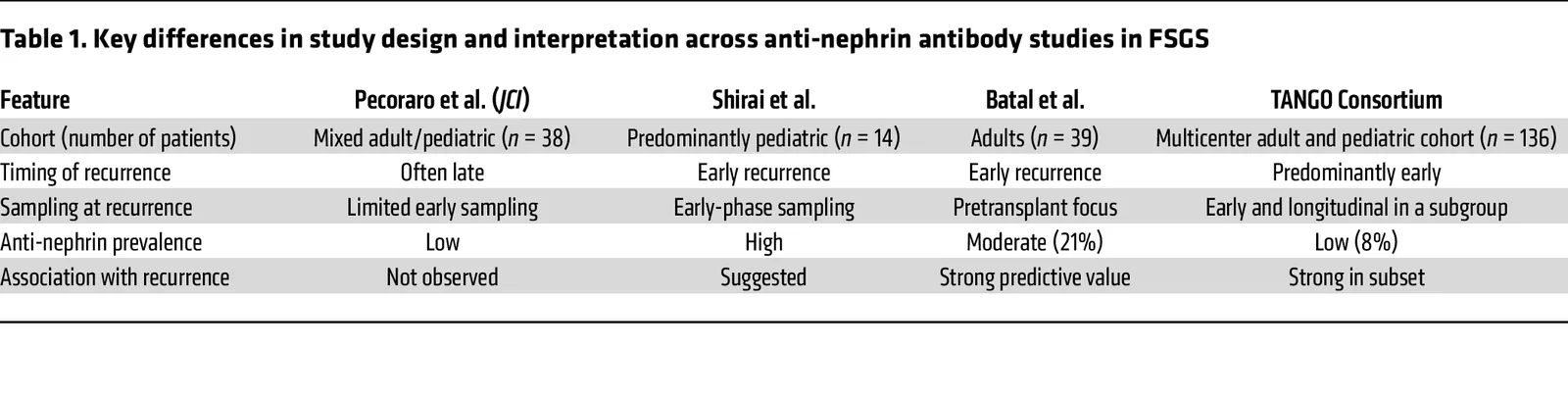
Key differences in study design and interpretation across anti-nephrin antibody studies in FSGS

## Version 1 11/16/2026

Electronic publication
